# Higher BMI is associated with higher expiratory airflow normalised for lung volume (FEF25–75/FVC) in COPD

**DOI:** 10.1136/bmjresp-2017-000231

**Published:** 2017-10-13

**Authors:** Eric Abston, Alejandro Comellas, Robert Michael Reed, Victor Kim, Robert A Wise, Roy Brower, Spyridon Fortis, Reinhard Beichel, Surya Bhatt, Joseph Zabner, John Newell, Eric A Hoffman, Michael Eberlein

**Affiliations:** 1 Department of Medicine, University of Iowa, Iowa City, Iowa, USA; 2 Division of Pulmonary, Critical Care and Occupational Medicine, University of Iowa, Iowa City, Iowa, USA; 3 Division of Pulmonary and Critical Care Medicine, University of Maryland School of Medicine, Baltimore, Maryland, USA; 4 Division of Pulmonary and Critical Care Medicine, Temple University, School of Medicine, Philadelphia, Pennsylvania, USA; 5 Division of Pulmonary and Critical Care Medicine, Johns Hopkins University, Baltimore, Maryland, USA; 6 Department of Electrical and Computer Engineering, University of Iowa, Iowa City, Iowa, USA; 7 The Iowa Institute for Biomedical Imaging, University of Iowa, University of Iowa, Iowa City, Iowa, USA; 8 Division of Pulmonary, Allergy and Critical Care Medicine, University of Alabama, Birmingham, Alabama, USA; 9 Department of Radiology, University of Iowa, Iowa, USA

**Keywords:** copd epidemiology, lung physiology

## Abstract

**Introduction:**

The obesity paradox in chronic obstructive pulmonary disease (COPD), whereby patients with higher body mass index (BMI) fare better, is poorly understood. Higher BMIs are associated with lower lung volumes and greater lung elastic recoil, a key determinant of expiratory airflow. The forced expiratory flow (25–75) (FEF_25–75_)/forced vital capacity (FVC) ratio reflects effort-independent expiratory airflow in the context of lung volume and could be modulated by BMI.

**Methods:**

We analysed data from the COPDGene study, an observational study of 10 192 subjects, with at least a 10 pack-year smoking history. Data were limited to subjects with BMI 20–40 kg/m^2^ (n=9222). Subjects were stratified according to forced expiratory volume in 1 s (FEV_1_) (%predicted)-quintiles. In regression analyses and Cox proportional hazard models, we analysed the association between BMI, the FEF_25–75_/FVC ratio, the imaging phenotype, COPD exacerbations, hospitalisations and death.

**Results:**

There was no correlation between BMI and FEV_1_(%predicted). However, a higher BMI is correlated with a higher FEF_25–75_/FVC ratio. In CT scans, a higher BMI was associated with less emphysema and less air trapping. In risk-adjusted models, the quintile with the highest FEF_25–75_/FVC ratio was associated with a 46% lower risk of COPD exacerbations (OR 0.54, p<0.001) and a 40% lower risk of death (HR 0.60, p=0.02), compared with the lowest quintile. BMI was not independently associated with these outcomes.

**Conclusions:**

A higher BMI is associated with lower lung volumes and higher expiratory airflows when normalised for lung volume, as quantified by the FEF_25–75_/FVC ratio. A higher FEF_25–75_/FVC ratio is associated with a lower risk of COPD exacerbations and death and might quantify functional aspects of the paradoxical effect of higher BMIs on COPD.

**Key messages**

- The obesity-paradox in COPD, whereby patients with higher body mass index (BMI) fare better, is poorly understood.
- In an ancillary study to COPDGene a higher BMI is associated with lower lung volumes and higher expiratory airflows when normalized for lung volume, as quantified by the FEF_25-75_/FVC-ratio and a higher FEF_25-75_/FVC-ratio is independently associated with a lower risk of COPD exacerbations and death.
- The FEF_25-75_/FVC-ratio quantifies functional aspects of the paradoxical effect of BMI on COPD and further understanding of the physiological mechanism could lead to novel non-pharmacological therapies based on the analogies to chest wall strapping.

## Introduction

There exists a poorly understood obesity paradox in chronic obstructive pulmonary disease (COPD),^1^ where obese patients with COPD tend to fare better than non-obese patients with similar degree of airflow obstruction.^2^ Observational studies show that over time obese patients with COPD experience lower mortality and fewer hospital admissions.^3 4^ Obesity has also been associated with lower mortality in patients with acute exacerbations.^5^

The mechanisms underlying this obesity paradox in COPD are unclear. Higher body mass index (BMI) in patients with COPD is associated with lower functional residual capacity (FRC)) and residual volume (RV),^6^ likely related to the mass effects of adipose tissue acting on the chest wall or abdomen.^7^ In addition to affecting the chest wall, higher BMI is associated with greater static lung elastic recoil, and in some studies with increased expiratory flow,^8–10^ as lung elastic recoil of the lung is the key determinant of maximal expiratory airflow.

The ratio of mid-vital capacity expiratory airflow (forced expiratory flow (25–75) (FEF_25–75_)) divided by the forced vital capacity (FVC) corresponds to effort-independent expiratory airflow adjusted for lung volume. We hypothesised that (1) a higher BMI is associated with a higher FEF_25–75_/FVC ratio and (2) that the FEF_25–75_/FVC ratio, as a possible functional correlate of the physiological effects of obesity, modulates the risk for COPD exacerbations and death.

## Methods

**Figure 1:**
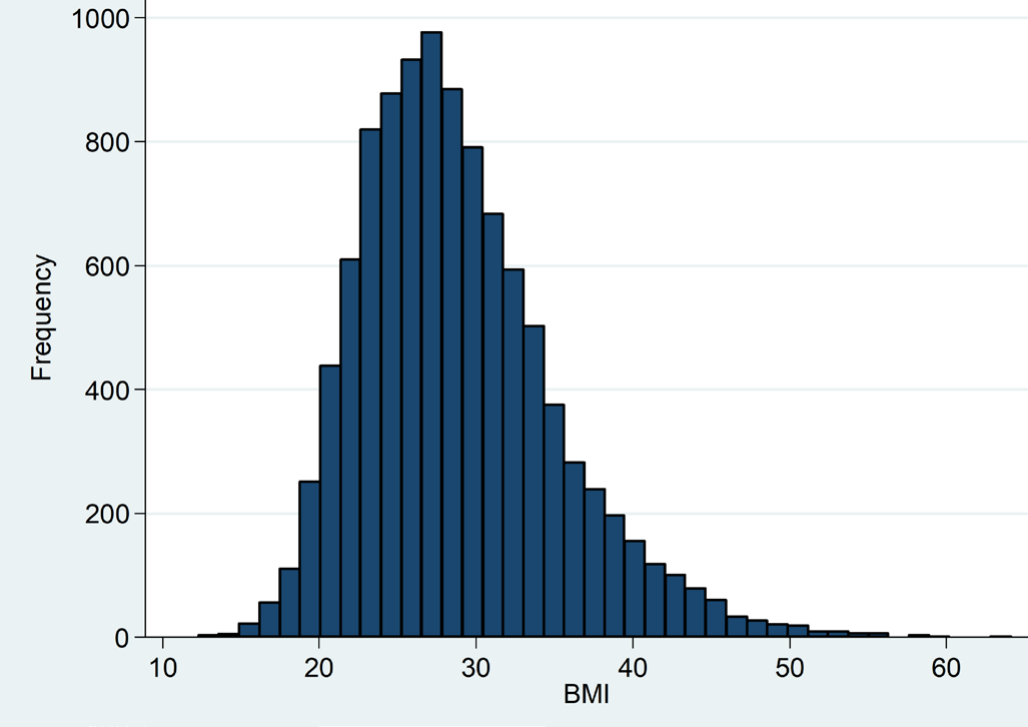
Histogram of the distribution of body mass index for the study population.

We evaluated data from the COPDGene study—an observational cohort study of 10 192 participants across 21 centres in the USA (2008–2011). Participants were non-Hispanic Whites and African-Americans with at least a 10 pack-year smoking history.^11^ Each participant provided informed written consent. The COPDGene protocol has been previously described and is available at www.copdgene.org.^11^ Methods pertinent to the data analysed in this study are as follows: subjects completed spirometry according to the American Thoracic Society standards.^11^ High-resolution CT scans were performed at full inspiration and at end exhalation. Quantitative measures of emphysema were defined as the percentage of lung volume on the inspiratory CT with attenuation less than −950 Hounsfield units (HU).^12^ Gas trapping was defined as the percentage of lung volume on the expiratory CT with attenuation less than −856 HU.^12^ The data were limited in this study to subjects with a BMI between 20 and 40 kg/m^2^ (n=9222) in order to limit the effects of spurious values and at physiological extremes of the BMI spectrum. Figure 1 shows a BMI histogram for the entire study population.

The study subjects were stratified according to forced expiratory volume in 1 s (FEV_1_) (%predicted)-quintiles. The relationship between BMI, CT-imaging phenotype (CT volumetry, emphysema and air trapping), spirometry (FEV_1_, FVC and FEV_1_/FVC) and the FEF_25-75_/FVC ratio for the entire study population and within each FEV_1_ (%predicted)-quintile was analysed by Spearman’s rank correlation coefficients (Spearman’s rho) and by using a fractional polynomial approach to evaluate for a possible non-linear association. We used logistic regression to evaluate the relationship between the FEF_25–75_/FVC ratio, BMI and the occurrence of COPD exacerbations. The outcome variable, COPD exacerbation, was examined as a binary variable. A univariate analysis was performed to assess for variables that were associated with COPD exacerbation at a threshold of p=0.1 as previously described,^13^ and those variables identified were then included in stepwise backward multivariate logistic models to adjust for confounders.

To evaluate the relationship between the FEF_25–75_/FVC ratio, BMI and the occurrence of COPD exacerbations in the study follow-up period, the COPDGene Longitudinal Follow-Up (LFU) dataset was utilised.^14^ The LFU dataset consists of telephone survey data obtained every 3–6 months after the initial study. Information on subjects including COPD exacerbations and hospitalisations was obtained. Follow-up COPDGene mortality data were analysed to evaluate the relationship between the FEF_25–75_/FVC ratio and mortality in the follow-up period. Cox proportional hazard models were used for the mortality analysis. We tested interactions between FEF_25–75_/FVC ratio and BMI, and the significance of differences between nested models was tested using the likelihood ratio test. Subjects with missing data were excluded from the respective analyses.

## Results

Patient demographics including metrics of disease severity, comorbid conditions, imaging parameters and spirometric values is shown in the table 1 in the online supplementary file 1, stratified by FEV_1_(%predicted)-quintiles. Comorbid conditions were more common in those with more severe COPD. BMI was not significantly different between FEV_1_-quintiles. All metrics of disease severity correlated with FEV_1_(%predicted)-quintiles.

10.1136/bmjresp-2017-000231.supp1 Supplementary file 1

**Figure 2:**
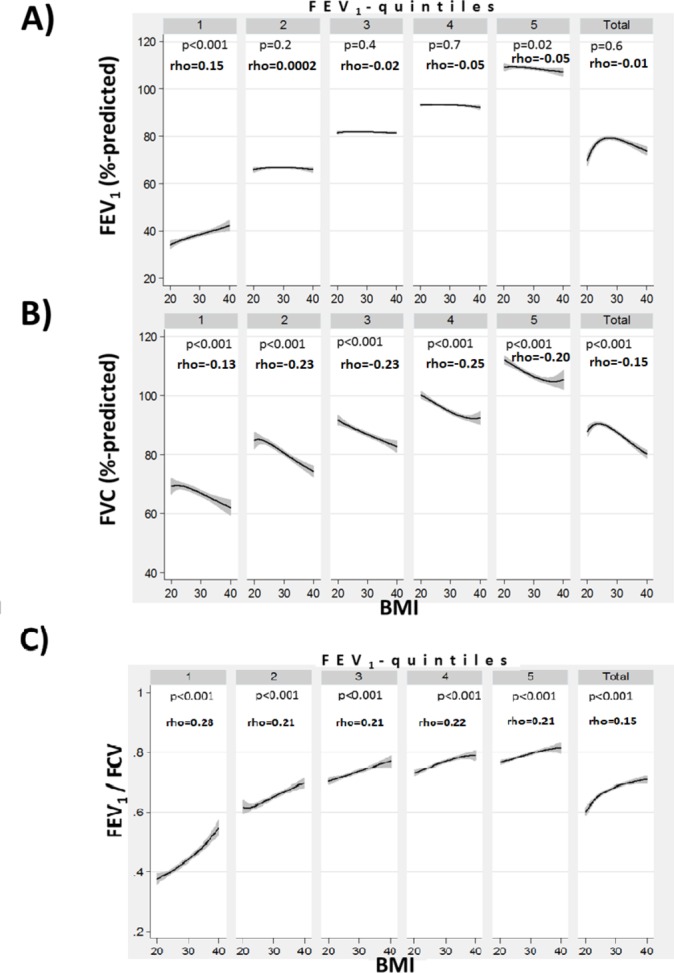
The relationship between body mass index (BMI) and per cent predicted forced expiratory volume in 1 s (FEV_1)_ (A) and per cent predicted forced vital capacity (FVC) (B) and the FEV_1_/FVC ratio (C), stratified by FEV_1_ quintiles and for the entire study population (BMI 20–40). Spearman’s rank correlation coefficients (Spearman’s rho) are shown and significance of the correlation is indicated by the corresponding p value.

**Figure 3:**
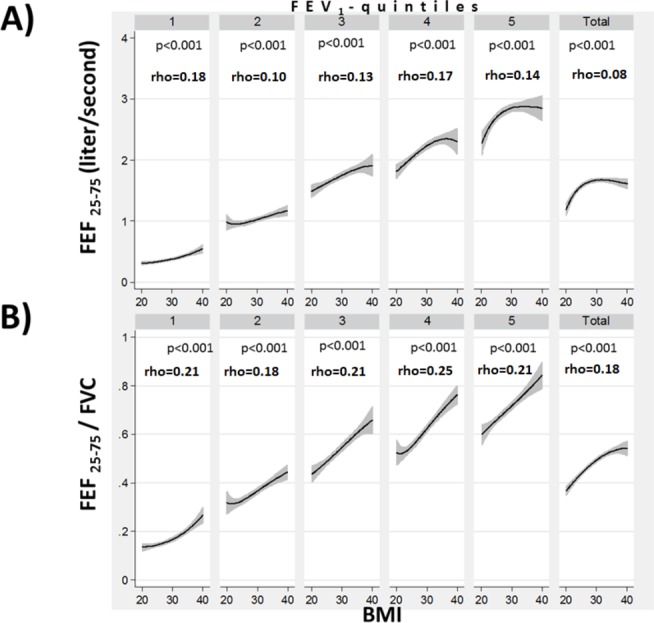
The relationship between body mass index (BMI) and forced expiratory flow (25-75) (FEF_25–75_) (A) and (FEF_25–75_)divided by forced vital capacity (FVC) (B), stratified by forced expiratory volume in 1 s (FEV_1_) quintiles and for the entire study population (BMI 20–40). Spearman’ s rank correlation coefficients (Spearman’s rho) are shown and significance of the correlation is indicated by the corresponding p value.

No consistent relationship between FEV_1_(%predicted) and BMI were found when analysing the entire sample (p=0.6), or within most FEV_1_(%predicted)-quintiles (figure 2A). However, a higher BMI was associated with lower FVC(%predicted) (figure 2B). Consequently, a higher BMI was associated with higher FEV_1_/FVC ratios, both in the whole study population and in each FEV_1_(%predicted)-quintile (p<0.001) (figure 2C). A higher BMI was associated with higher FEF_25–75_, both in the entire study population and in each quintile of FEV_1_ (p=0.001) (figure 3A). There was a positive association between the FEF_25–75_ /FVC ratio and BMI, both in the entire study population and in each quintile of FEV_1_(%predicted) (p=0.001) (figure 3B).

**Figure 4:**
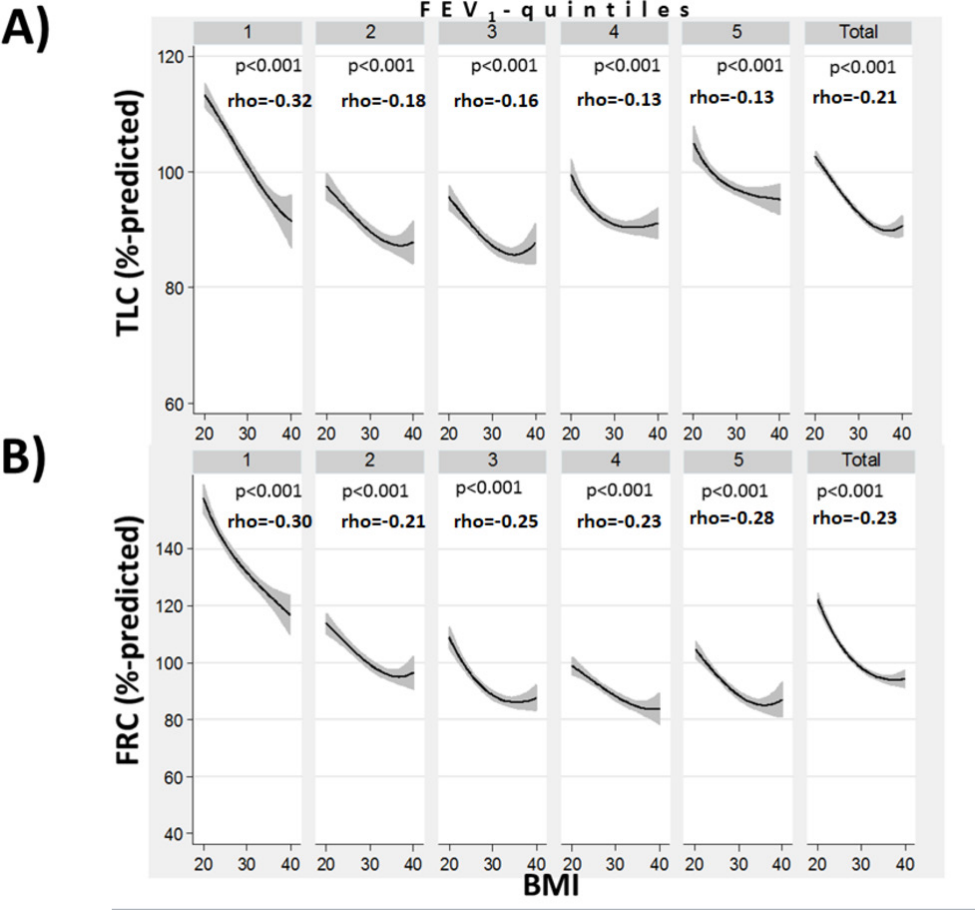
The relationship between body mass index (BMI) and total lung capacity (TLC) (A) and functional residual capacity (FRC) (B), stratified by forced expiratory volume in 1 s (FEV_1_) quintiles and for the entire study population (BMI 20–40). Spearman’s rank correlation coefficients (Spearman’s rho) are shown and significance of the correlation is indicated by the corresponding p -value.

**Figure 5:**
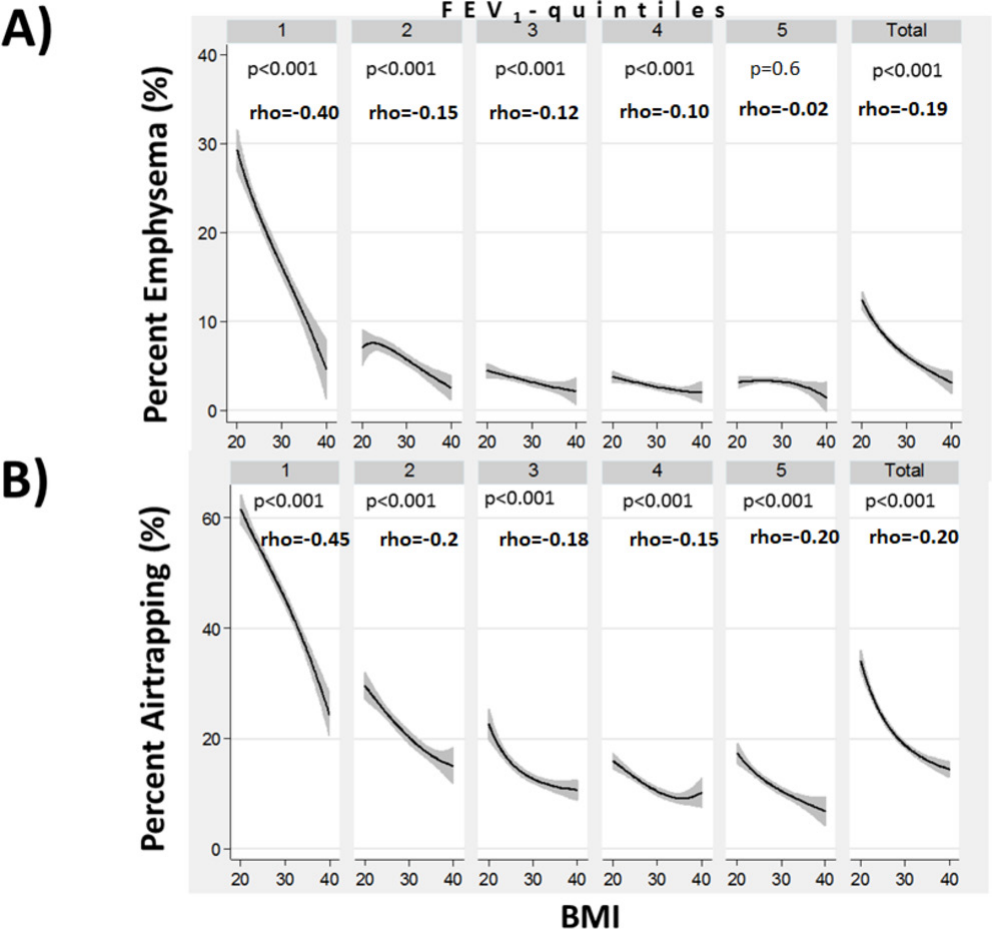
The relationship between body mass index (BMI) and per cent emphysema (A) and per cent air trapping (B), stratified by forced expiratory volume in 1 s (FEV_1_) quintiles and for the entire study population (BMI 20–40). Spearman’s rank correlation coefficients (Spearman’s rho) are shown and significance of the correlation is indicated by the corresponding p value.

A higher BMI was associated with lower total lung capacity (TLC) (%predicted) and functional residual capacity (FRC) (%predicted) derived from CT volumetry (figure 4). A higher BMI correlated inversely with per cent emphysema (p<0.001) within most FEV_1_(%predicted)-quintile and for the entire study population (figure 5A). The slope of the line fitting this association was much steeper in the FEV_1_(%predicted)-quintile with the lowest FEV_1_. This indicates that comparatively smaller differences in BMI correlated with much larger differences in emphysema in subjects with more severe disease than in quintiles with a more normal FEV_1_. A higher BMI correlated inversely with per cent air trapping (p<0.001) on expiratory CT scans within each FEV_1_(%predicted)-quintile and for the entire study population (figure 5B). This reduction in air trapping with increasing BMI (from 20 to 40) was strongest in the FEV_1_(%predicted)-quintile with the lowest FEV_1_.

As the FEF_25–75_ /FVC ratio could reflect the physiological impact of BMI on lung function we next evaluated the association between FEF_25–75_/FVC ratio, BMI and clinical outcomes in COPD. In unadjusted models a higher FEF_25–75_ /FVC ratio was associated with a lower risk of self-reported COPD exacerbations at study entry and a lower occurrence of COPD exacerbations during the study follow-up period (table 1A). Furthermore, a higher FEF_25–75_/FVC ratio was associated with a lower risk of hospitalisations and a lower risk of death during the study follow-up period. When adjusting for BMI the FEF_25–75_/FVC ratio remained independently associated with the above clinical outcomes, whereas BMI itself did not remain consistently associated with these outcomes (table 1B). In comprehensive risk-adjusted models, a higher FEF_25–75_/FVC ratio remained independently associated with a lower risk of COPD exacerbations, hospitalisation and mortality in the follow-up period.

**Table 1: The association of FEV 25-75 / FVC ratio and BMI with COPD outcomes:** 

Parameter	**(A)** Model 1, univariate			**(B)** Model 2, multivariate adjusted (as indicated in legend)		
Exacerbation* history†	**OR**	**95%**	**p**	**OR**‡	**95%**	**p**
FEF_25–75_/FVC-ratio Quintile 1 versus 5	0.11	0.09 to 0.14	**<0.001**	0.55	0.21 to 1.40	0.2
BMI Quintile 1 versus 5	1.28	1.10 to 1.50	**0.002**	1.08	0.65 to 1.79	0.7
Exacerbation* on follow up§	**OR**	**95%**	**p**	**OR**‡	**95%**	**p**
FEF_25–75_/FVC ratio Quintile 1 versus 5	0.28	0.25 to 0.32	**<0.001**	0.54	0.39 to 0.73	**<0.001**
BMI Quintile 1 versus 5	1.1	0.97 to 1.25	0.14	1.13	0.892 to 1.39	0.2
Hospitalised**	**OR**	**95%**	**p**	**OR**¶	**95%**	**p**
FEF_25–75_/FVC ratio Quintile 1 versus 5	0.09	0.07 to 0.21	**<0.001**	0.41	0.27 to 0.63	**<0.001**
BMI Quintile 1 versus 5	0.97	0.79 to 1.19	0.8	1.30	0.94 to 1.79	0.1
**Mortality††**	**HR**	**95%**	**p**	**HR**¶	**95%**	**p**
FEF_25–75_/FVC ratio Quintile 1 versus 5	0.17	0.13 to 0.23	**<0.001**	0.60	0.39 to 0.94	**0.02**
BMI Quintile 1 versus 5	0.62	0.50 to 0.78	**<0.001**	0.74	0.54 to 1.01	0.06

*Exacerbation analysis is stratified according to exacerbation yes/no.

†Exacerbation data from first study visit.

‡Adjusted for: BMI, age at enrolment, history of severe exacerbations, chronic bronchitis, asthma, American Thoracic Society (ATS) pack-year smoking, current smoking, fume exposure at work, gastro-oesophageal reflux disease, congestive heart failure, sleep apnoea, history of blood clots, high blood pressure, Modified Medical Research Council dyspnoea scale, St. George’s Respiratory Questionnaire score, forced expiratory volume in 1 s (%predicted) and 6 min walk distance.

§Exacerbation data from longitudinal follow-up data set.

¶Adjusted for: BMI, pack-year smoking, current smoking, oxygen use, per cent emphysema and Body mass index, airflow Obstruction, Dyspnoea and Exercise score.

**Hospitalisation analysis is stratified according to yes/no.

††Complete mortality data were available for 7534 subjects.

BMI, body mass index; FEF_25–75_, forced expiratory flow (25–75); FVC, forced vital capacity.

## Discussion

In a large cohort of current and former smokers, we found that that FEV_1_ was largely unaffected by BMI. However, when analysing expiratory airflow in the context of the corresponding lung volumes via the FEF_25–75_ /FVC ratio, a positive association between the FEF_25–75_/FVC ratio and BMI became evident. A higher FEF_25–75_/FVC ratio would either predict higher elastic recoil or greater small airway sizes for the same lung volume, and consistent with this we found higher BMI was associated with lesser emphysema and less air trapping. A higher FEF_25–75_ /FVC ratio was associated with a lower risk of COPD exacerbations and death and might be a parameter that quantifies possible physiological effects associated with a higher BMI on COPD outcomes.

Classically, FEV_1_ has been used to quantify the disease severity in COPD. However, FEV_1_ was not modulated by obesity-related changes in lung function in previous studies,^9^ and similarly we did not find a consistent association between BMI and FEV_1_ in the current study. However, we found a positive correlation between BMI and FEF_25–75_ and an inverse relation between BMI and FVC. A misclassification of the severity of airflow obstruction by stratifying according to FEV1(%predicted) could have affected the study results, as a reduction of lung volumes due to a restrictive process, like obesity, can add to the reduction in FEV_1_ from an obstructive disease.^15^ Thus, the reduced lung volumes from obesity might lead to an overestimation of the severity of the obstructive process. We performed a sensitivity analysis in which we adjusted the FEV_1_ (%predicted) by the total lung capacity (TLC) (% predicted), as derived from CT scans at full inspiration. We created FEV_1_(%predicted)_adjusted_=(FEV1(%predicted)/TLC (%predicted)). We then restratified subjects according to quintiles of FEV_1_(%predicted)_adjusted_. This sensitivity analysis showed similar results (see figures 1 and 2 in the online supplementary file 1) and a higher BMI remained associated with a higher FEF_25–75_/FVC ratio, lesser emphysema and lesser air trapping.

Jere Mead developed a similar ratio (FEF_25–75_/FVC multiplied by lung elastic recoil) to reflect the size of the airway structure in relation to the lung volume.^16^ He used this ratio to express the physiological variation in the geometry of the tracheobronchial tree and lung parenchyma due to different patterns of growth among both genders, which he termed dysanapsis.^16^ Thus, the higher FEF_25–75_/FVC ratio with increasing BMIs we found in this study would either predict higher elastic recoil or larger airway sizes for the same lung volume. A key finding of this study was that, as predicted by the association with a higher FEF_25–75_/FVC ratio, a higher BMI correlated inversely with per cent air trapping on expiratory CT scans (figure 5B). Air trapping is correlated with the closing volume of small airways and an isolated deflating effect of obesity should not affect the closing volume of small airways. Imaging studies in COPD have demonstrated that small conducting airways narrow and disappear before the onset of emphysematous disease.^17^ If it is correct that widespread narrowing and loss of smaller conducting airways precedes the onset of emphysematous destruction, then a possible beneficial effect of obesity on small airway function could reduce the risk for progression to emphysema. Similar to observations made in the Multiethnic Study of Atherosclerosis lung study,^18^ we found that a higher BMI was associated with less emphysema (figure 5). This could suggest that obesity modulates small airway function in COPD.

The effects of obesity on pulmonary physiology have been carefully characterised both in healthy subjects and in those with COPD.^9^ The most prominent aspect of obesity-induced changes in the respiratory system is the reduction in expiratory reserve volume (ERV), followed by more modest reductions in other lung volumes.^10^ Obesity also reduces lung compliance and increases lung elastic recoil in several studies.^8 19^ There are analogies between the effects of obesity on the respiratory system and chest wall strapping (CWS).^20–28^ CWS is a technique to restrict chest and abdominal wall motion during respiration to force the lung to operate at lower lung volumes.^20–28^ CWS causes a decrease in lung volumes, an increase in expiratory flows and airway conductance, a decrease in lung compliance and an increase in lung elastic recoil.^20–28^ CWS reduces TLC by ~35% with similar changes in vital capacity, FRC and similarly to obesity ERV is reduced the most by 50%.^20–28^ Lung elastic recoil is increased by on average 180%.^20^ CWS increases mid-vital capacity maximum expiratory flows to on average 150% of prestrapped rates.^20^ The increased lung elastic recoil from CWS could increase the radial traction on small airways via the interdependence between airways and parenchyma.^20 29 30^ This increase radial traction could dilate small airways resulting in lower closing volumes and higher airway conductance.^20 29 30^ Consistent with this possible mechanism, a study of normal subjects and in subjects with mild to moderate COPD showed that CWS increased the number of small airways detectable via an automated CT airway segmentation algorithm.^31–33^ Furthermore, transplanting significantly oversized donor lungs into a recipient with a smaller chest cavity is conceptually similar to CWS and was associated with higher mid-vital capacity expiratory airflows and higher FEV_1_/FVC ratios.^20 34–37^ The FEV_1_/FVC ratio is conceptually also a ‘dysanapsis ratio’. Similar to the FEF_25–75_/FVC ratio, the FEV_1_/FVC ratio varied with BMI (figure 2C). Thus, a higher BMI could be associated with a normal FEV_1_/FVC ratio, even in the presence of obstructive airways disease. This may explain some of the observations regarding the ‘GOLD–Unclassified Smokers’ or ‘preserved ratio impaired spirometry’ phenotype that is characterised by a reduced FEV_1_ with a preserved FEV_1_/FVC ratio.^38^ Supporting this possibility, the ‘GOLD–Unclassified Smokers’ phenotype was associated with increased BMIs.^38^

This study was limited in several aspects. Due to the cross-sectional nature of the study, we cannot determine a causal relationship between BMI and the imaging phenotype (lower TLC, FRC, less emphysema and air trapping). While the data is suggestive that obesity could have a CWS-like effect of increasing lung elastic recoil, we do not have measurements of lung elastic recoil or expiratory airflows at isovolume conditions. The FEF_25–75_/FVC ratio has been developed as a surrogate measure of airway size relative to lung size or lung dysanapsis. While FVC can be an appropriate surrogate of lung size in normal lungs, it can be reduced due to forced exhalation in subjects with COPD and emphysema, where FVC can be significantly lower than the slow vital capacity. In a sensitivity analysis, we have generated a ‘dysanapsis’ ratio that instead of FVC included TLC (derived from CT volumetry), as a measurement of lung size. There is close linear relationship between the FEF_25–75_/FVC ratio and the FEF_25–75_/TLC ratio (Spearman’s rho was 0.97, p<0.0001, see figure 3 in the online supplementary file 1). Also, BMI itself is an imperfect metric of adiposity, and different fat distribution patterns can result in varying respiratory effects with similar BMI. As gender can affect both fat distribution patterns and airway structure, we performed a sensitivity analysis stratified by gender, which showed similar results (see figure 4 in the online supplementary file 1). In this study, we limited the BMI to between 20 and 40, which excluded 970 subjects (approximately 10% of the study population). When we analysed the entire study cohort, the overall results were not different (see figures 5 and 6 in the online supplementary file 1).

There appears to be a plateau effect to the obesity-induced changes in lung function at the extremes of BMI. Mild to moderate obesity shares the greatest similarities with lung function changes observed with CWS, whereas extreme obesity, especially when FRC or ERV are reduced below certain thresholds can be associated with worsening lung function.^39^ In this study, the effect of BMI on FEF_25–75_ and FEF_25–75_/FVC seems to be more pronounced from BMI 20 to 30, then from BMI 30 to 40 (figure 3). Also, obesity is a complex chronic condition with varied patterns of fat depositions as well as many systemic and behavioural associations extending beyond mechanical effects of adipose tissue.^9 40^ Finally, even though multivariable modelling was used to account for possible confounding, it is understood that variables not available or missing variables in this data set may result in residual confounding.

In conclusion, increased BMI is associated with lower lung volumes, lesser emphysema and air trapping. The FEF_25–75_/FVC ratio, as a dysanapsis ratio, seems to quantify the physiological impact of obesity on the COPD phenotype and is independently associated with COPD exacerbations and mortality. BMI affects the COPD phenotype in a manner that has similarities to CWS, which could provide a possible mechanistic basis for aspects of the BMI paradox seen in COPD.
