# Supplementary material for: Higher BMI is associated with higher expiratory airflow normalised for lung volume (FEF25–75/FVC) in COPD
Source: BMJ Open Respir Res. 2017 Oct 13;4(1):e000231. doi: 10.1136/bmjresp-2017-000231 (PMC5652498; doi:10.1136/bmjresp-2017-000231)
Supplement: Supplementary file 1 [file bmjresp-2017-000231supp001.pdf]

ONLINE SUPPLEMENT to

A higher BMI is associated with higher expiratory airflow normalized for lung volume (FEF_{25-75}/FVC) in COPD

Eric Abston MD[1], Alejandro P Comellas MD[1.2], Robert M Reed MD[3], Victor Kim MD[4], Robert A Wise MD[5], Roy G Brower MD[5], Spyridon Fortis MD[1,2], Reinhard R Beichel PhD[1,6,7], Surya P Bhatt MD[8], Joseph Zabner MD[1.2], John D Newell MD[7,9], Eric A Hoffman PhD[1,6,7,9], Michael Eberlein MD PhD[1,2]

[1]Department of Medicine, [2]Division of Pulmonary, Critical Care and Occupational Medicine, University of Iowa - Iowa City, IA/US, [3] Division of Pulmonary and Critical Care Medicine, University of Maryland School of Medicine - Baltimore, MD/US, [4] Division of Pulmonary and Critical Care Medicine, Temple University School of Medicine - Philadelphia, PA/US, [5] Division of Pulmonary and Critical Care Medicine, Johns Hopkins University - Baltimore, MD/US, [6] Department of Electrical and Computer Engineering and [7]The Iowa Institute for Biomedical Imaging, University of Iowa, Iowa City, IA/US [8]Division of Pulmonary, Allergy and Critical Care Medicine, University of Alabama - Birmingham, AL/US [9]Department of Radiology, University of Iowa- Iowa City, IA/US

Keywords:, COPD, body mass index, obesity, elastic recoil, expiratory airflow, Chest wall strapping

Corresponding author:
Michael Eberlein MD PhD
Division of Pulmonary, Critical Care and Occupational Medicine
University of Iowa Hospitals and Clinics
200 Hawkins Drive, C 33 GH
Iowa City, IA 52242
Phone: 319-356-1265; Fax: 319-353-6406; E-mail: michael-eberlein@uiowa.edu

Online Supplemental Table 1:

FEV1 (%predicted) quintile	1	2	3	4	5			
Variable	Mean±Std						ANOVA	Bonferroni 1 vs 5
Age	64±8	61±9	59±9	58±9	57±9		<0.001	<0.001
Gender (% female)	40%	51%	46%	47%	44%		<0.001	0.652
Race White (%)	80%	70%	67%	64%	60%		<0.001	<0.001
BMI	27.7±4.8	29.0±4.9	28.7±4.7	28.3±4.7	27.7±4.3		<0.001	0.337
BODE	4.1±1.6	1.4±1.3	0.6±1.0	0.4±0.9	0.3±0.7		<0.001	<0.001
MMRC	2.6±1.2	1.6±1.4	1.0±1.3	0.7±1.2	0.6±1.1		<0.001	<0.001
Gold Stage	3.1±0.9	1.1±1.4	0.3±1.0	0.1±0.5	0.0±0.4		<0.001	<0.001
SGRQ	48±19	32±22	22±20	17±18	14±16		<0.001	<0.001
Smoking (packyears)	56±29	49±25	43±24	39±21	36±20		<0.001	<0.001
Current Smoker (%)	30%	50%	60%	60%	60%		<0.001	<0.001
Occupational Exp	80%	70%	60%	60%	60%		<0.001	<0.001
Meters Walked (6 Min)	1060±380	1310±350	1410±340	1500±350	1550±340		<0.001	<0.001
O2 (L/min)	1.02±1.85	0.13±0.76	0.04±0.46	0.01±0.22	0.01±0.18		<0.001	<0.001
CHF	6%	4%	2%	2%	1%		<0.001	<0.001
Sleep Apnea	53%	53%	43%	41%	37%		<0.001	<0.001
GERD	31%	29%	26%	21%	18%		<0.001	<0.001
Asthma	66%	48%	35%	28%	19%		<0.001	<0.001
Hypertension	52%	49%	42%	35%	34%		<0.001	<0.001
Chronic Bronchitis	30%	24%	16%	13%	11%		<0.001	<0.001
Blood Clots	5%	6%	4%	3%	2%		<0.001	<0.001
Exacerbation history								
Severe Exacerbation	28%	14%	7%	5%	3%		<0.001	<0.001
TLC (L)	6.3±1.5	5.4±1.4	5.2±1.3	5.4±1.3	5.7±1.4		<0.001	<0.001
TLC % Pred	105±18	92±17	89±15	93±14	99±15		<0.001	<0.001
FRC (L)	4.5±1.3	3.3±0.9	2.9±0.8	2.9±0.8	2.9±0.8		<0.001	<0.001
FRC % Pred	140±32	104±26	94±22	92±20	94±21		<0.001	<0.001
Emphysema (%)	18.2±13.4	5.7±7.5	2.9±4.3	2.5±3.3	2.8±3.5		<0.001	<0.001
GasTrapping (%)	50±18	23±16	14±12	12±10	12±10		<0.001	<0.001
FEV1 % Pred	37±11	65±6	81±4	93±3	108±8		<0.001	<0.001
FVC %Pred	67±	81±	87±	96±	108±		<0.001	<0.001
FEV1 /FVC	0.42±	0.64±	0.73±	0.76±	0.79±		<0.001	<0.001
FEF 25-75 (L/sec)	0.37±22	1.06±0.60	1.83±0.83	2.39±0.93	3.12±1.12		<0.001	<0.001
FEF 25-75 / FVC	0.16±0.12	0.35±0.21	0.52±0.26	0.60±0.25	0.69±0.25		<0.001	<0.001

Online Supplemental Table 1: Study parameters displayed by quintile of FEV1 as mean ± standard deviation. Quintiles were compared by ANOVA, and post hoc analysis with Bonferroni analysis of quintile 1 vs quintile 5 shown.

Online Supplemental Figure 1:

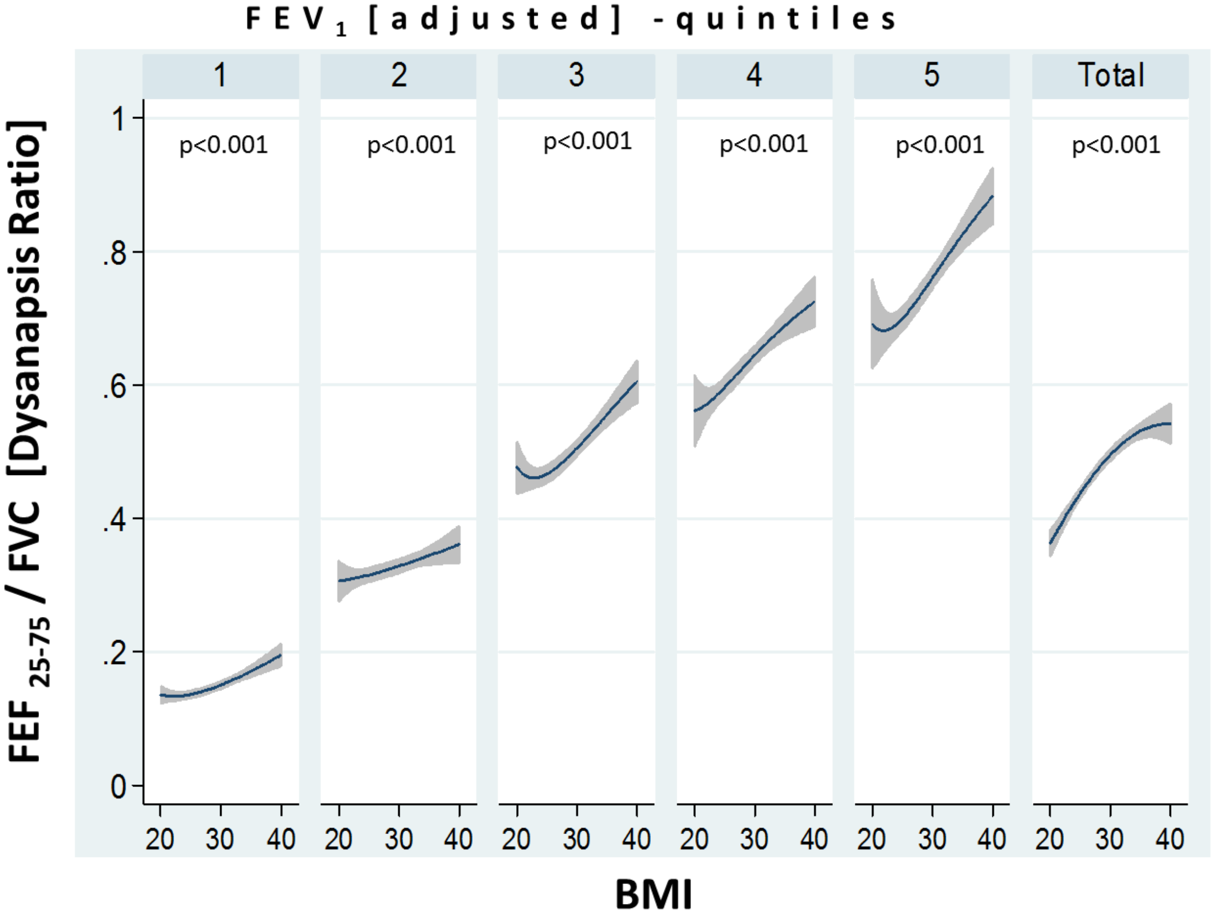

Online Supplemental Figure 1: The relationship between BMI and Forced Expiratory Flow (25-75) divided by Forced Vital Capacity [Dysanapsis Ratio], stratified by FEV_1 [adjusted] quintiles and for the entire study population. Univariate linear regression analysis shows a significant relationship for each quintile and in total ($p<0.001$). FEV_1 [adjusted] = FEV_1(%predicted) / TLC(%predicted).

Online Supplemental Figure 2:

A)

B)

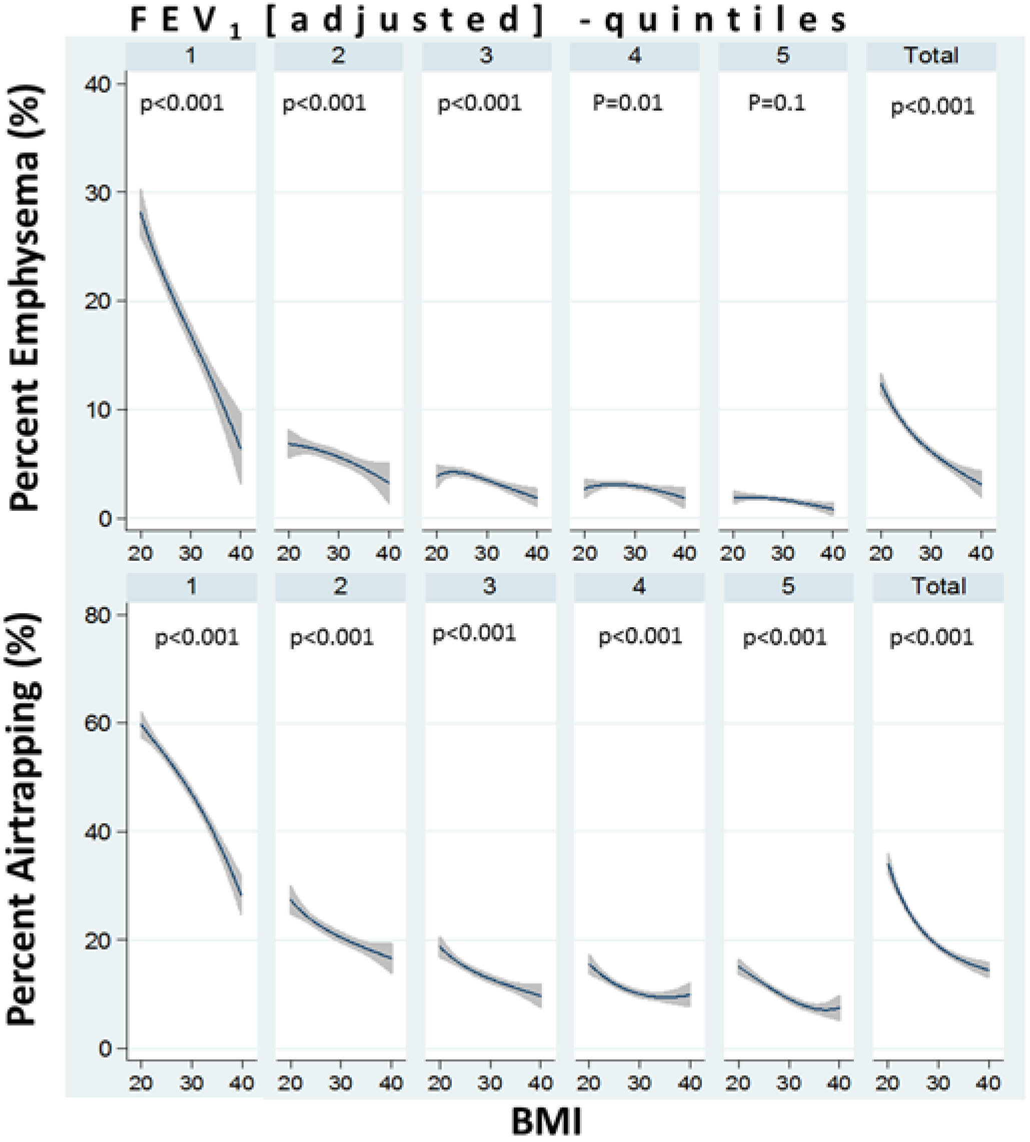

Online Supplemental Figure 2: The relationship between BMI and Percent emphysema (A) and percent air trapping (B), stratified by FEV_1 [adjusted] quintiles and for the entire study population. Univariate linear regression analysis shows a significant relationship for each quintile and in total for both percent air trapping and percent emphysema ($p<0.001$). FEV_1 [adjusted] = FEV_1(%predicted) / TLC(%predicted).

Online Supplemental Figure 3:

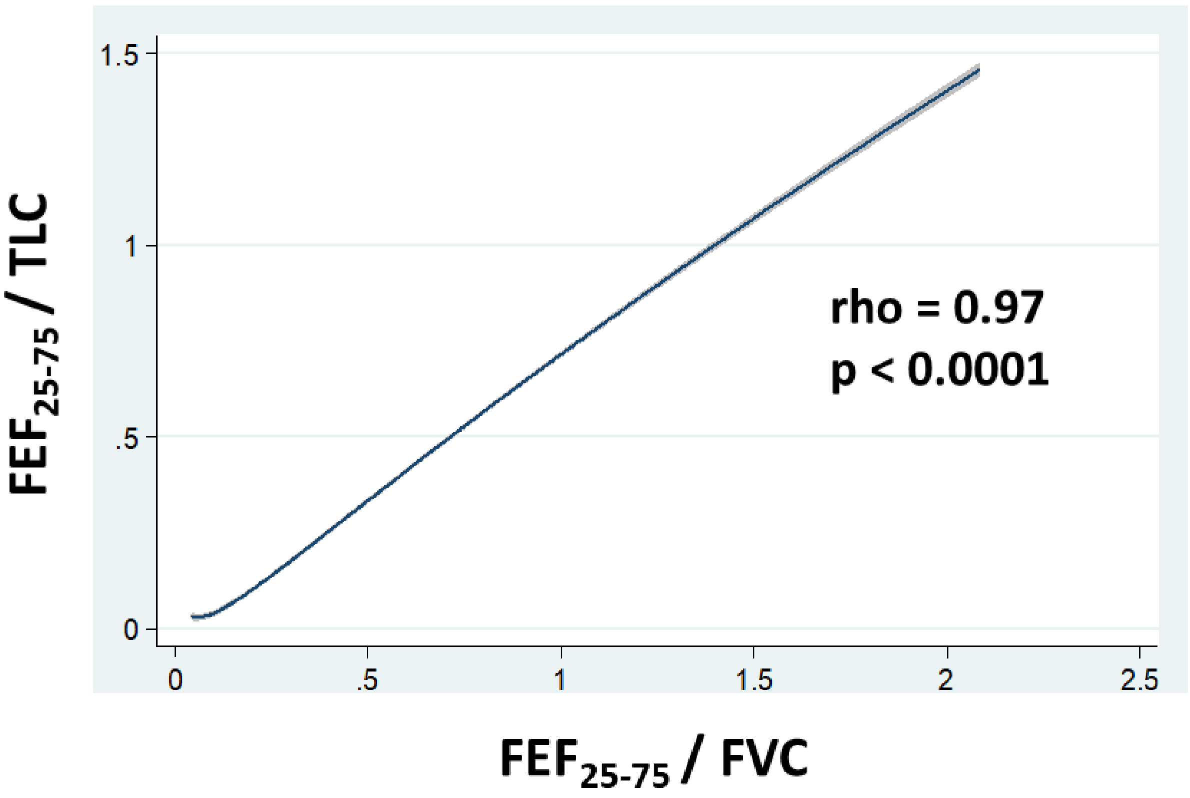

Online Supplemental Figure 3: The relationship between Forced Expiratory Flow (25-75) [$FEF_{25\text{-}75}$] divided by Forced Vital Capacity [FVC] and Forced Expiratory Flow (25-75) [$FEF_{25\text{-}75}$] divided by Total Lung Capacity [TLC] (derived from Computer tomographic volumetry). Spearmans rank correlation coefficient (spearman's rho) is shown and significance of the correlation is indicated by the corresponding p-value.

Online Supplemental Figure 4:

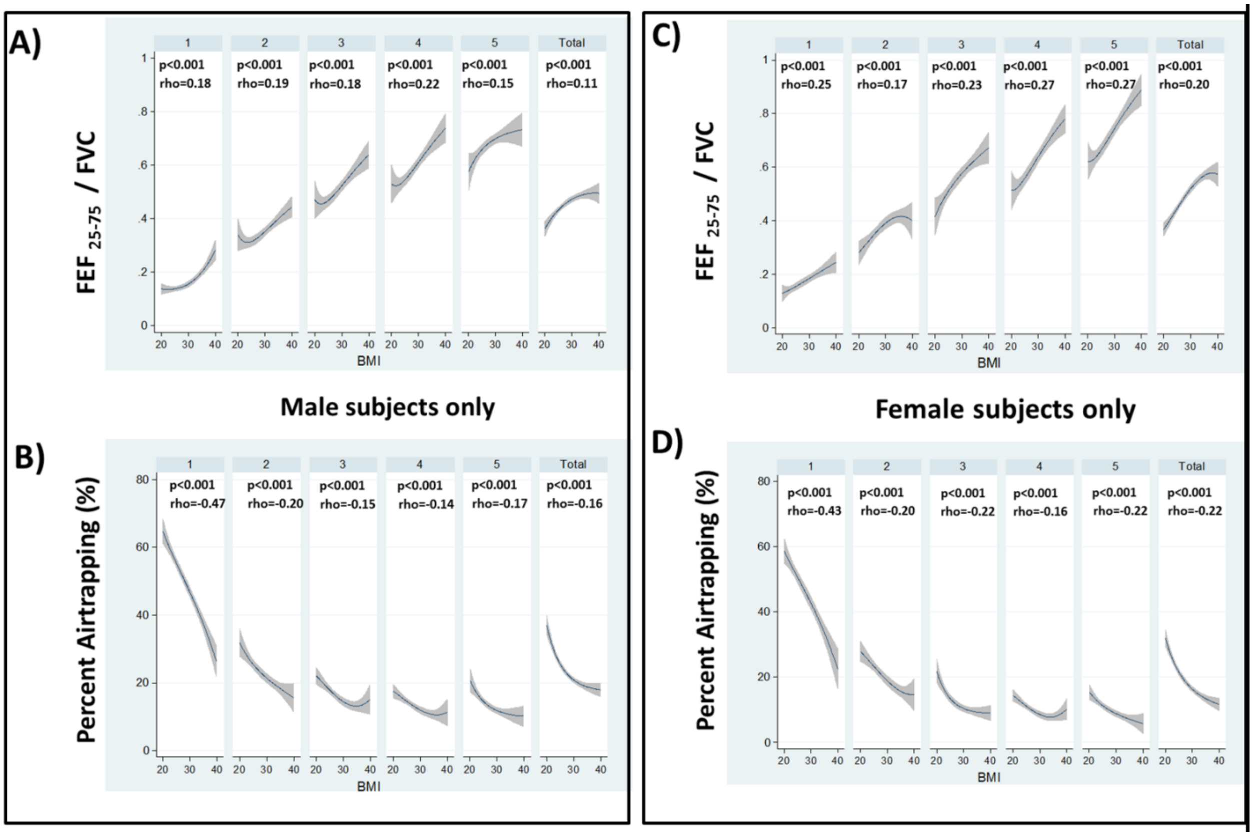

Online Supplemental Figure 4: The relationship between BMI and Forced Expiratory Flow (25-75) divided by Forced Vital Capacity and Airtrapping is shown stratified by FEV_1 quintiles and for the entire study population. These relationshops are shown for male (A and B) and female (C and D) study subjects as indicated. Spearmans rank correlation coefficients (spearman's rho) are shown and significance of the correlation is indicated by the corresponding p-value.

Online Supplemental Figure 5:

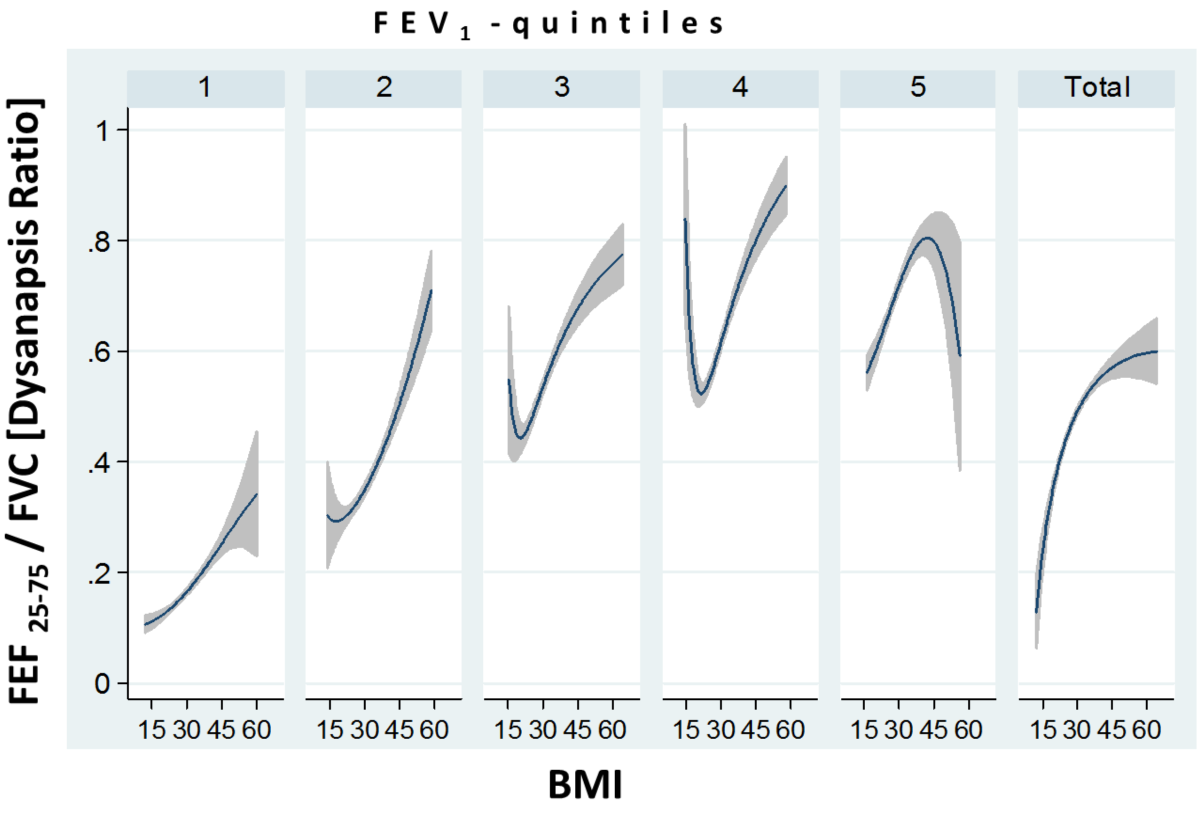

Online Supplemental Figure 5: The relationship between BMI and Forced Expiratory Flow (FEF $_{25\text{-}75}$) divided by Forced Vital Capacity [Dysanapsis Ratio], stratified by FEV_1 (%-predicted) quintiles and for the entire study population (no BMI limitations).

Online Supplemental Figure 6:

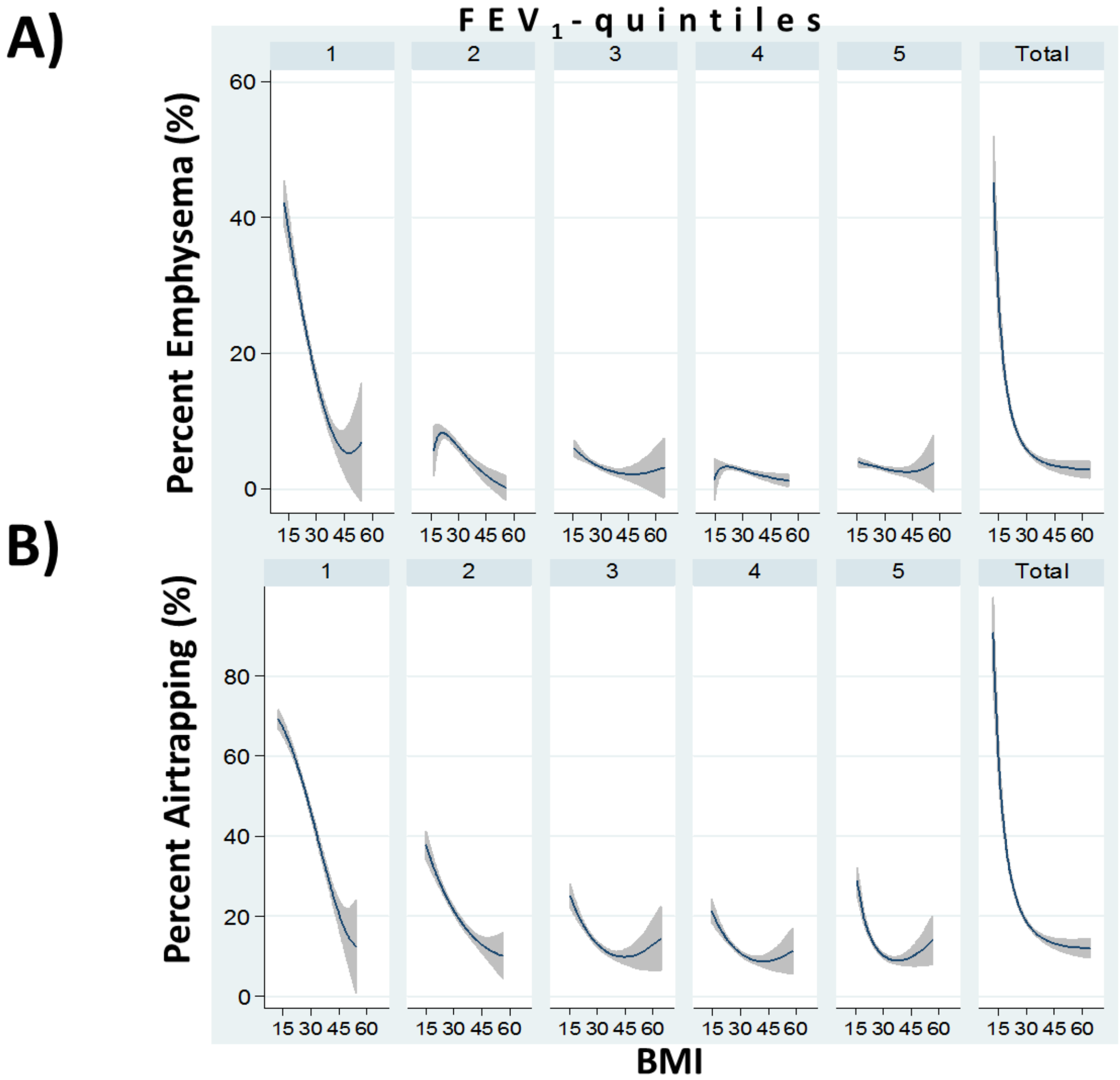

Online Supplemental Figure 6: The relationship between BMI and Percent emphysema (A) and percent air trapping (B), stratified by FEV_1 (%-predicted) quintiles and for the entire study population (no BMI limitations).
